# Spontaneous Splenic Rupture Unveiled: A Non-traumatic Case Associated With Infective Endocarditis

**DOI:** 10.7759/cureus.45664

**Published:** 2023-09-21

**Authors:** Fábio Viveiros, Cristina Silva, Ana Cristina Rodrigues, Rui Escaleira, Alberto Midões

**Affiliations:** 1 General Surgery, Unidade Local de Saúde do Alto Minho (ULSAM), Viana do Castelo, PRT

**Keywords:** aortic valve replacement, splenectomy, hypovolemic shock, infective endocarditis, spontaneous splenic rupture

## Abstract

Spontaneous splenic rupture (SSR) is a rare and potentially life-threatening condition often associated with trauma. However, SSR can occur without evident trauma, presenting unique diagnostic challenges. We present a case report of a 32-year-old postpartum female who experienced sudden-onset abdominal pain and was diagnosed with SSR. Despite the absence of trauma, she exhibited hypovolemic shock, requiring rapid intervention. Diagnostic imaging, including CT scans, revealed a substantial splenic laceration, which led to an emergent splenectomy. The patient’s postoperative course was complicated by infective endocarditis (IE) with aortic involvement, elucidated as the underlying cause of SSR. The patient underwent aortic valve replacement, received antibiotic therapy, and achieved a successful recovery. This case underscores the importance of early recognition, timely intervention, and collaboration among diverse medical specialties in managing SSR cases. Furthermore, it highlights the potential link between SSR and IE, emphasizing the meaning of considering infectious etiologies even in non-traumatic scenarios. Early identification of the underlying cause is crucial for effective management and positive patient outcomes in cases of SSR.

## Introduction

Typically, splenic rupture is linked to traumatic incidents. Nonetheless, there are instances where it occurs without any traumatic event. This occurrence is referred to as an atraumatic splenic rupture or spontaneous splenic rupture (SSR). Its occurrence is uncommon, accounting for approximately 0.1-0.5% of all reported cases of splenic ruptures [1].

SSR exhibits a higher prevalence in males, with an average age of onset of around 42 years old [2]. A diverse array of disease processes and benign conditions have been linked to SSR. These conditions include a range of categories, including infectious diseases such as infectious mononucleosis, tuberculosis, infective endocarditis (IE), and zoonoses. Additionally, SSR can be associated with neoplastic conditions, congenital factors like hemangiomas and benign cysts, autoimmune disorders, hematologic issues, as well as various miscellaneous causes [3].

This condition carries the potential for fatality, and patients may exhibit a diverse range of clinical symptoms and physical indicators. The majority of patients commonly describe experiencing pain in the left upper quadrant (LUQ) of the abdomen. In more severe instances, hypotension and neurological dysfunction might also manifest. Diagnosing this condition presents challenges, as splenic rupture is often omitted from the list of potential causes of abdominal pain in the absence of trauma. This oversight can lead to SSR evolving into a life-threatening situation with ease [3,4].

Here, we present a case report of a spontaneous splenic rupture, followed by a review of the literature on this subject.

## Case presentation

A 32-year-old female arrived at the surgical emergency room, complaining of a sudden, intense abdominal pain that had begun a few hours prior. The pain was widespread throughout her abdomen and radiated to her back. The pain emerged abruptly while the patient was at rest in bed. Notably, the patient had experienced odynophagia and fever three days prior to presenting at the hospital. The patient denied nausea, vomiting, diarrhea, or any other accompanying symptoms.

Fifteen days prior to this episode, the patient had given birth through a normal delivery process. The labour proceeded without complications, and there was no evidence of manual fundal pressure. Regarding her medical history, there were no notable details aside from two uneventful pregnancies.

Upon examination, the patient presented with a body temperature of 37.6 ºC, exhibiting hypotension (BP: 86/31 mmHg) and tachycardia (140 beats/min). Although awake and conscious, the patient appeared pallid, and the capillary perfusion time exceeded three seconds. She also displayed tachypnea. During the abdominal examination, tenderness was observed throughout the abdomen, accompanied by guarding. Abdominal sounds were absent, and signs of peritoneal irritation were evident.

The initial blood tests upon admission indicated the following results: a hemoglobin level of 5.6 g/dl, a white blood cell count of 32,780/mm^3^, with 83% neutrophils; the platelet count was measured at 332,000/mm^3^. Additionally, the CRP level was recorded at 14.85 mg/dl, and the serum lactate level was measured to be 3.4 g/dl. Other blood test parameters were within normal ranges, including coagulation biomarkers.

**Figure 1:**
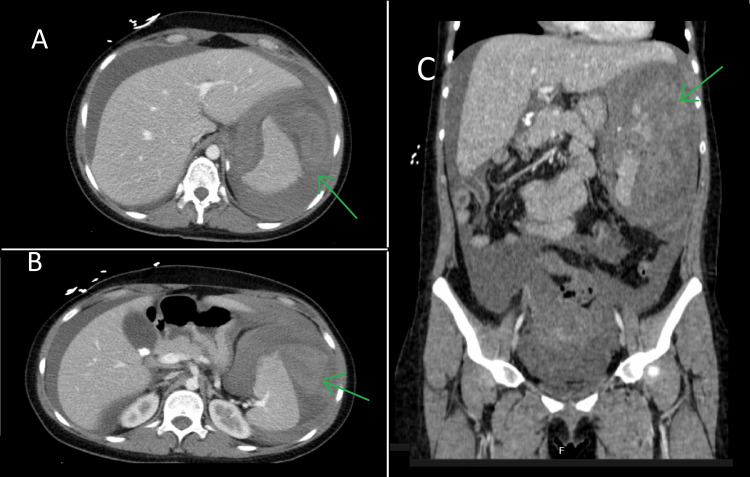
(A and B) Axial and (C) coronal section of an abdominal CT. It can be seen the presence of contrast material extravasation, along with the detection of free peritoneal fluid and hyperdense elements within the LUQ. CT: computed tomography.

The massive transfusion protocol was promptly initiated, and the initial resuscitation procedures were undertaken. The abdominal CT scan (Figure 1) revealed a sizable subcapsular lesion situated on the lateral aspect of the spleen. This lesion exhibited heterogeneous content, displaying areas of spontaneous hyperdensity that strongly indicated the presence of a significant hematoma that was creating a mass effect. The CT scan disclosed a profound laceration along the anterior surface within the middle third of the spleen. This laceration was notable for an arterial extravasation of contrast material, signifying active bleeding. Accompanying these findings was a notable volume of free peritoneal fluid, which occupied both the upper and lower quadrants. Notably, the pelvic region of the peritoneal cavity contained a hyperdense component, suggestive of the presence of blood clots. There was no evidence of pneumoperitoneum, and no additional findings were observed.

Based on these findings, the patient was directly transferred to the OR for an exploratory laparotomy. Initial assessment of the abdominal cavity unveiled a substantial hemoperitoneum, accompanied by the predominant accumulation of blood clots in the LUQ as well as the pelvic region. Active bleeding emanated from the inferior pole of the spleen, evidencing a capsular rupture. In response, a splenectomy was expeditiously carried out.

Subsequent to the splenectomy, a peritoneal lavage procedure was conducted, which yielded an estimated blood loss of approximately 3 liters.

Following the surgical procedure, the patient was admitted to the intensive care unit (ICU). Subsequent investigation led to the identification of IE affecting a native aortic valve, coupled with severe acute aortic failure. In light of this diagnosis, a decision was made to transfer the patient to a specialized referral hospital with a cardiothoracic surgery unit. There, the patient underwent an aortic valve replacement procedure, wherein a biological prosthesis was implanted. Simultaneously, a comprehensive antibiotic regimen consisting of vancomycin and gentamicin was administered over a span of six weeks. This regimen aimed to address the underlying infection, despite the absence of identification of any specific causative organism in the blood cultures.

Histological examination revealed a spleen of normal size with evidence of rupture. No additional indications of underlying pathology were detected.

The patient’s clinical progression showcased an exceptional and uneventful recovery. This positive evolution culminated in the patient’s discharge at the conclusion of the six-week period. Importantly, both the echocardiogram and heart function evaluations returned to normal ranges. No long-term complications were acknowledged.

## Discussion

A spleen rupture constitutes a critical medical emergency due to its significant potential for fatality. The primary cause of spleen rupture is abdominal trauma, predominantly blunt trauma [5]. Nevertheless, instances of non-trauma-related spleen rupture are infrequent. This case stood out due to its swift progression and life-threatening nature, considering the non-traumatic scenario.

In this specific case, the patient arrived at the surgical emergency room displaying evident signs of hypovolemic shock, which were subsequently indicative of hemorrhagic shock based on the hemoglobin levels. Immediate stabilization and resuscitation measures facilitated the acquisition of an abdominal CT scan prior to the exploratory laparotomy. This proactive approach proved highly advantageous, enabling a more efficient surgical strategy and effective management of the hemorrhage.

Following the resolution of the surgical emergency and successful patient stabilization, the prompt identification of the underlying cause of SSR - in this instance, IE accompanied by severe acute aortic failure - facilitated the early adoption of a cardiothoracic surgical intervention. This timely and accurate diagnosis further enabled the implementation of the appropriate treatment regimen, ultimately leading to the patient’s complete recovery despite the gravity of her life-threatening condition.

The success of this care can also be attributed to the effective collaboration among various medical specialties, including general surgeons, intensive care physicians, and cardiothoracic surgeons. This underscores the pivotal role of teamwork in managing intricate cases, highlighting its significance in achieving positive outcomes.

As per the existing literature, the presenting features of SSR typically manifest as abrupt and widespread abdominal or chest pain. These symptoms often exhibit a degree of vagueness and occasional confusion. Unfortunately, this characteristic places them at the peril of being erroneously linked to more prevalent conditions like cardiac events or gastrointestinal illness. The repercussions of such misinterpretations carry significant gravity due to the rapid progression toward hemorrhagic shock, necessitating urgent intervention [6,7].

While abdominal ultrasound serves as a diagnostic tool for detecting splenic rupture, it is noteworthy that numerous studies have highlighted CT scanning as the optimal choice in cases where splenic rupture is suspected. This preference is primarily due to the heightened sensitivity of CT scans [6,8].

The investigation in this case report led us to an IE. Typically, instances of splenic rupture in the context of IE occur after the affected valve has been replaced. The patient also did not belong to a high-risk group for IE, such as intravenous drug users. Embolization’s occurrence is variable, ranging between 20% and 40% in cases of IE, and it stands as a significant contributor to mortality, particularly in the form of cerebrovascular accidents [9,10]. Comparatively, the incidence of splenic embolism is notably lower, with reports ranging from 5% to 12%. Several potential mechanisms underlie splenic rupture in IE, including simple infarction, abscess formation following embolization, and posteriorly suppurative necrosis [9]. In this particular case, it is pertinent to consider fundal pressure during vaginal delivery as an alternative explanation for splenic rupture, even though there was no evidence of this procedure in the patient's history, as it can lead to late presentation due to pseudoaneurism formation.

Splenic ruptures can be addressed through either operative (surgical) procedures or nonoperative approaches such as angioembolization. However, in our patient’s case, her condition did not permit the application of a nonoperative strategy. This specific approach necessitated stabilization and subsequent transfer to another healthcare facility, as our center lacked an interventional radiology team capable of conducting the procedure on that particular day.

## Conclusions

SSR is an uncommon occurrence, typically seen in tandem with IE post-diagnosis. However, in this instance, the patient displayed an SSR of undetermined origin. Recognizing SSR early and initiating prompt treatment is pivotal for survival, given its life-threatening potential. Equally crucial is early identification of its underlying cause, as it not only forestalls subsequent incidents but also paves the way for a potential complete recovery. This case report underlines the imperative of considering IE as a potential cause of SSR in cases where the etiology remains obscure.
